# Presence of transient hydronephrosis immediately after surgery has a limited influence on renal function 1 year after ileal neobladder construction

**DOI:** 10.1186/s12894-017-0263-x

**Published:** 2017-08-31

**Authors:** Takuma Narita, Shingo Hatakeyama, Takuya Koie, Shogo Hosogoe, Teppei Matsumoto, Osamu Soma, Hayato Yamamoto, Tohru Yoneyama, Yuki Tobisawa, Takahiro Yoneyama, Yasuhiro Hashimoto, Chikara Ohyama

**Affiliations:** 1 Department of Urology, Hirosaki University Graduate School of Medicine, 5 Zaifu-cho, Hirosaki, 036-8562 Japan; 2 Department of Advanced Transplant and Regenerative Medicine, Hirosaki University Graduate School of Medicine, Hirosaki, Japan

**Keywords:** eGFR, Hydronephrosis, Ileal neobladder, Radical cystectomy, Renal function

## Abstract

**Background:**

Urinary tract obstruction and postoperative hydronephrosis are risk factor for renal function deterioration after orthotopic ileal neobladder construction. However, reports of relationship between transient hydronephrosis and renal function are limited.

We assess the influence of postoperative transient hydronephrosis on renal function in patients with orthotopic ileal neobladder construction.

**Methods:**

Between January 2006 and June 2013, we performed radical cystectomy in 164 patients, and 101 received orthotopic ileal neobladder construction. This study included data available from 64 patients with 128 renal units who were enrolled retrospectively. The hydronephrosis grade of each renal unit scored 0–4. The patients were divided into 4 groups according to the grade of hydronephrosis: control, low, intermediate, and high. The grade of postoperative hydronephrosis was compared with renal function 1 month and 1 year after surgery.

**Results:**

There were no significant differences in renal function before surgery between groups. One month after surgery, the presence of hydronephrosis was significantly associated with decreased renal function. However, 1 year after urinary diversion hydronephrosis grades were improved significantly, and renal function was comparable between groups. Postoperative hydronephrosis at 1 month had no significant influence on renal function 1 year after ileal neobladder construction. Limitations include retrospective design, short follow-up periods, and a sample composition.

**Conclusions:**

The presence of transient hydronephrosis immediately after surgery may have limited influence on renal function 1 year after ileal neobladder construction.

## Background

Urinary diversion after radical cystectomy is mandatory for muscle-invasive bladder cancer patients, and it should ensure protection of the upper urinary tract. Orthotopic ileal neobladder construction following cystectomy has evolved in an attempt to restore anatomy and function as close as possible to the preoperative state. Several risk factors have been reported for the postoperative decline in renal function [1–4]. Recent retrospective studies suggested that risk factors associated with renal function decline are urinary tract obstruction [2] and postoperative hydronephrosis [4]. However, the progression of hydronephrosis and renal function after ileal neobladder construction are not well defined, and there are few reports describing the relationship between transient hydronephrosis and renal function following radical cystectomy and orthotopic ileal neobladder construction. Moreover, the association between transient hydronephrosis and renal function was not studied. Therefore, the aim of this study was to investigate the effect of postoperative hydronephrosis on renal function in patients with orthotopic ileal neobladder construction. Specifically, we compared the grade of postoperative hydronephrosis with renal function 1 month and 1 year after surgery.

## Methods

### Ethics statement

This study was performed in accordance with the ethical standards of the Declaration of Helsinki, and approved by an ethics review board of Hirosaki University School of Medicine (The authorization number: 2015–047). The participants in this study provide their verbal informed consent when hospitalized, and it was recorded in medical chart. Pursuant to the provisions of the ethics committee and the ethic guideline in Japan, written consent was not required in exchange for public disclosure of study information in the case of retrospective study. The study information was open for the public consumption at http://www.med.hirosaki-u.ac.jp/~uro/html/IRB/IRBdoc.html.

### Patient selection

Between January 2006 and June 2013, we performed radical cystectomy in 164 patients, and 101 received orthotopic ileal neobladder construction. Of these, 34 patients without serum creatinine levels and/or computed tomography (CT) imaging within 1 year after surgery as well as three with unilateral nephroureterectomy were excluded. As a result, 64 patients with 128 of renal-units were enrolled in this retrospective study. Tumor stage and grade were assigned according to the 2009 TNM classification of the Union of International Cancer Control [5].

### Evaluation of hydronephrosis grade and classification

**Fig. 1:**
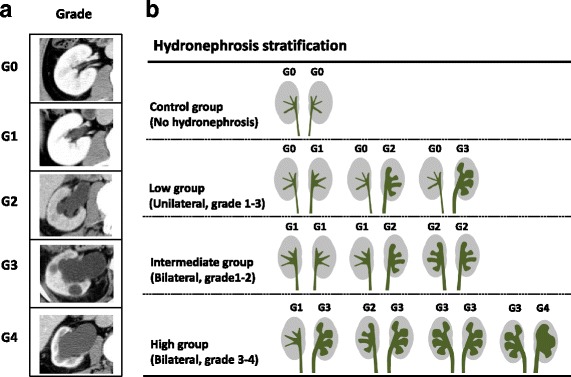
Hydronephrosis grade and stratification. **a**. The hydronephrosis grade of each renal unit was evaluated by computed tomography (CT) imaging, and scored according to hydronephrosis grading scale: grade 0, no dilatation (G0); grade 1, pelvic dilatation only (G1); grade 2, mild caliceal dilatation (G2); grade 3, severe caliceal dilatation (G3); and grade 4, renal parenchymal atrophy (G4). **b**. Patients were stratified into 4 groups according to hydronephrosis grade and status (unilateral or bilateral): no hydronephrosis in bilateral kidneys (control group), unilateral hydronephrosis (low group), bilateral hydronephrosis with G1 or G2 (intermediate group), and bilateral hydronephrosis with G3 or G4 on either side (high group)

The hydronephrosis grade of each renal unit was evaluated by CT imaging, and scored according to the hydronephrosis grading scale: grade 0, no dilatation (G0); grade 1, pelvic dilatation only (G1); grade 2, mild caliceal dilatation (G2); grade 3, severe caliceal dilatation (G3); grade 4, renal parenchymal atrophy (G4) (Fig. 1a), as described previously [6, 7]. It was measured by single urologist who was blinded to the outcomes. The patients were also stratified into 4 groups according to hydronephrosis grade and status (unilateral or bilateral): no hydronephrosis in bilateral kidney (control group), unilateral hydronephrosis (low), bilateral hydronephrosis with G1 or G2 (intermediate), and bilateral hydronephrosis with G3 or G4 on either side (high) (Fig. 1b). The grade of postoperative hydronephrosis was compared with renal function 1 month and 1 year after surgery.

### Evaluation of renal function and clinical parameters

Renal function was evaluated using estimated glomerular filtration rate (eGFR) using a modified version of the abbreviated Modification of Diet in Renal Disease Study formula [8]: eGFR mL/min/1.73 m^2^ = 194 × sCr^−1.094^ × age^−0.287^ (×0.739, if female). Each patient was evaluated using preoperative and postoperative eGFR after 1 month and 1 year.

We analyzed the variables including age, gender, Eastern Cooperative Oncology Group Performance Status (ECOG-PS), history of cardiovascular disease, hypertension, diabetes mellitus, renal function (eGFR) clinical and pathological stage, blood loss, operative duration, postoperative complications, and tumor recurrence. We defined pyelonephritis as a positive urine culture and tenderness with fever (axillary temperature > 38.5 °C). Repeated postoperative episodes (at least 2 or more) of acute pyelonephritis were recorded as postoperative complications. Hypertension was defined as any antihypertensive medications intake or preoperative systolic and diastolic blood pressure measurements of >140 and >90 mmHg, respectively. Diabetic patients were defined as those who met the relevant diagnostic criteria, required glycemic control, and/or those with a history of type 2 diabetes.

### Surgical procedures

All patients underwent radical cystectomy, orthotopic ileal neobladder construction, and lymphadenectomy procedures. The basic procedure was identical regardless of the surgeon [9, 10]. Orthotopic ileal reservoir construction was performed as described previously [11, 12]. Key points of our procedures were that 1) resected a 40-cm of ileal segment approximately 20 cm proximal to the ileocecal valve, 2) ileal segment loop was arranged in a U shape, 3) an anti-reflux procedure was not performed in ureteroileal anastomosis.

### Patient follow-up

Ureteral stents were removed 1 week after surgery under radiographic guidance. An 18-F urethral catheter was removed 3 weeks after orthotopic ileal reservoir construction under radiographic guidance. We performed CT 1 month and 1 year after surgery as a routine work, and patients were discharged 4–6 weeks after surgery. Each patient was assessed every 3 months using ultrasonography to monitor for hydronephrosis; serum electrolytes, blood urea nitrogen, serum creatinine, and liver function were also measured. Subsequent formation of uretero-intestinal stricture was suspected and investigated when hydronephrosis was worsening. CT was performed every 6–12 months for the early detection of tumor recurrence. Urethroscopic examination and urine cytology was performed at 3-month intervals for 2 years.

### Statistical analysis

Statistical analyses of the clinical data were performed using SPSS ver. 19.0 (SPSS, Inc., Chicago, IL, USA) and GraphPad Prism 5.03 (GraphPad Software, San Diego, CA, USA). Categorical variables were compared using Fisher’s exact test or the χ^2^ test. Quantitative variables were expressed as mean with standard deviation or median with interquartile ratio (IQR). The differences between groups were compared statistically using Student’s *t*-test for normal distribution. Mann–Whitney *U*-test was used for the differences between groups with non-normal distribution. The Wilcoxon matched-pairs signed-rank test was used for matched pairs showing non-normal distribution. *P* values of <0.05 were considered to be statistically significant.

Risk factors for an eGFR <60 mL/min/1.73 m^2^ were identified using univariate and multivariate analyses with the logistic regression model, and odds ratios (ORs) with 95% confidence intervals (CI) were calculated after controlling simultaneously for potential confounders. Variables included in the models were age (>65.5 years), gender, history of cardiovascular disease, hypertension, type 2 diabetes, neoadjuvant chemotherapy, pathological T stage (>pT2), pathological lymph nodes involvement, postoperative complications, tumor recurrence, operative duration (>294 min), blood loss (>1380 g), hydronephrosis stratification (>low), and eGFR (<60 mL/min/1.73 m^2^).

## Results

The clinicopathological characteristics and distributions of the patients are presented in Table 1. A total of 64 patients underwent radical cystectomy and orthotopic ileal neobladder construction. The median age of this cohort was 65.5 years. Sixty-one patients (95%) received 2 cycles of platinum-based neoadjuvant chemotherapy, and cystectomy was done within 1 month after neoadjuvant chemotherapy. There were no patients required clean intermittent catheterization or indwelling catheter after the surgery. One patient required urethral bougie only once because of neobladder-urethral anastomotic stricture.

**Table 1:** Clinical and pathological patient characteristics

	All	Hydronephrosis stratification at 1 M				*P value*
		Control	Low	Intermediate	High	
n	64	12	11	27	14	
Age^a^	66 (61–71)	63 (55–69)	69 (60–73)	67 (59–71)	69 (63–75)	*0.401* ^*b*^
Gender (Male/Female), n=	48 / 16					
ECOG-PS	0.0 (0–0)	0.0 (0–0)	0.0 (0–0)	0.0 (0–0)	0.0 (0–0)	*1.000* ^*b*^
Past history, n=						
Cardiovascular disease	6 (9%)	1 ((%)	1 (9%)	3 (11%)	1 (7%)	*1.000* ^*c*^
Hypertension	21 (33%)	3 (25%)	3 (27%)	11 (41%)	4 (29%)	*0.765* ^*c*^
Diabetes	9 (14%)	1 (8%)	2 (18%)	5 (19%)	1 (7%)	*0.729* ^*c*^
Neoadjuvant chemotherapy, n=	61 (95%)	11 (92%)	11 (100%)	26 (96%)	13 (93%)	*0.882* ^*c*^
Clinical T stage^a^	2 (2–3)	3 (2–3)	2 (2–3)	2 (2–3)	3 (2–3)	*0.580* ^*b*^
Pathological T stage^a^	1 (0–2)	2 (0–2)	1 (0–2)	2 (1–2)	2 (0–2)	*0.578* ^*b*^
Pathological N+, n=	2 (3%)	0 (0%)	0 (0%)	1 (4%)	1 (7%)	*1.000* ^*c*^
eGFR^a^						
Before surgery	73 (66–87)	77 (75–97)	84 (73–91)	72 (65–88)	69 (62–73)	*0.062* ^*b*^
1 month after surgery	60 (53–74)	71 (57–82)	73 (60–88)	58 (52–65)	56 (43–72)	*0.029* ^*b*^
1 year after surgery	65 (56–74)	69 (62–76)	63 (53–78)	63 (53–74)	69 (61–75)	*0.524* ^*b*^
Hydronephrosis grades^a^						
Before surgery	0 (0–0)	0 (0–1)	0 (0–0)	0 (0–0)	0 (0–0)	*0.348* ^*b*^
1 month after surgery	2 (0–2)	0 (0–0)	1 (0–2)	2 (2–2)	3 (3–3)	*< 0.001* ^*b*^
1 year after surgery	0 (0–1)	0 (0–0)	0 (0–1)	0 (0–0)	1 (0–1)	*0.021* ^*b*^
Complications (any grades), n=	16 (25%)	2 (17%)	3 (27%)	10 (37%)	1 (7%)	0.213^*c*^
Complications (Clavien Grade > 3), n=	2 (2%)	0 (0%)	1 (9%)	1 (4%)	0 (0%)	0.261^*b*^
Operative duration^a^ (min)	294 (266–323)	281 (237–303)	277 (263–300)	322 (273–356)	280 (265–307)	*0.015* ^*b*^
Blood loss^a^ (g)	1380 (1034–2302)	1345 (576–2211)	1400 (1070–2465)	1470 (926–2350)	1430 (1095–2359)	*0.914* ^*b*^
Recurrence (within 1 year), n=	2 (3%)	0 (0%)	2 (18%)	0 (0%)	0 (0%)	*0.813* ^*c*^

^a^median (Q1-Q3)

Q1, first quartile; Q3, third quartile;^b^ Kruskal–Wallis test;^c^ Fisher’s exact test

Postoperative eGFR 1 year after surgery was significantly lower than preoperative eGFR (*P* < 0.001, Wilcoxon matched-pairs signed rank test); the median decrease in 1-year eGFR was 12% in all patient. (control group: 10.8%, low group: 24.2%, intermediate group: 11.0%, high group: 0.0%).

**Fig. 2:**
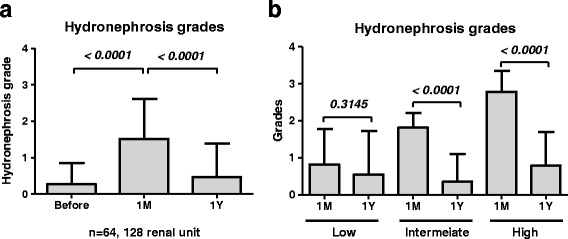
Preoperative and postoperative hydronephrosis grades. **a**. The overall hydronephrosis grades in 128 renal units were increased significantly 1 month after surgery (*P* < 0.001), but improved significantly 1 year after surgery (*P* < 0.001). **b**. The hydronephrosis grades in the low group did not changed significantly (*P* = 0.3145). The hydronephrosis grades in the intermediate and high groups were significantly increased 1 month after surgery (*P* < 0.0001), but improved significantly 1 year after surgery (*P* < 0.001). Statistical analyses were performed using Wilcoxon matched-pairs signed rank test

The preoperative and postoperative hydronephrosis grades of 128 renal units are shown in Fig. 2a. The median [first quartile (Q1)–third quartile (Q3)] preoperative, 1-month, and 1-year hydronephrosis grades were 0 (0–0), 2 (0–2), and 0 (0–1), respectively. The overall hydronephrosis grades in 128 renal units were increased significantly at 1 month after surgery, which improved significantly 1 year after surgery (Fig. 2a, *P* < 0.001, Wilcoxon matched-pairs signed rank test).

The patients were divided into 4 groups according to their hydronephrosis grade 1 month after surgery. The method used for stratification is shown in Fig. 1b. The numbers of patients in the control, low, intermediate, and high group were 12, 11, 27, and 14, respectively. There were no significant differences in patient background before surgery between the groups, except for operative duration (Table 1, *P* = 0.015, Kruskal–Wallis test). The hydronephrosis grades in the intermediate and high groups were increased significantly 1 month after surgery, which improved significantly 1 year after surgery (Fig. 2b, *P* < 0.001, Wilcoxon matched-pairs signed rank test).

**Fig. 3:**
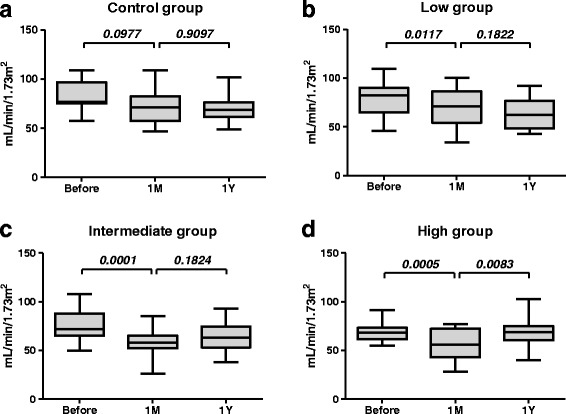
Preoperative and postoperative eGFR. **a**. No significant changes were observed in renal function before surgery and 1 month after surgery (*P* = 0.0977) or 1 month and 1 year after surgery (*P* = 0.9097) in the control group. **b and c**. The presence of mild hydronephrosis 1 month after surgery was significantly associated with renal function decline in the low (*P* = 0.0117) and intermediate (*P* = 0.0001) groups. Renal function became stable 1 year after surgery. **d**. The presence of severe hydronephrosis 1 month after surgery was significantly associated with a decline in renal function in the high group (*P* = 0.0005). However, renal function recovered significantly 1 year after surgery (*P* = 0.0083). Statistical analyses were performed using Wilcoxon matched-pairs signed-rank test

**Fig. 4:**
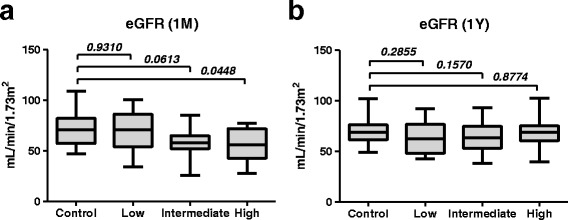
Intergroup postoperative eGFR differences compared with control. **a**. One month after surgery, postoperative eGFR was significantly lower in the high group. **b**. One year after surgery, postoperative eGFR was not significantly different between the high and control groups. Statistical analyses were performed using Mann–Whitney *U* test

In the control group, there were no significant changes in renal function before, or 1 month and 1 year after surgery (Fig. 3a). In contrast, the presence of hydronephrosis 1 month after surgery was significantly associated with a decline in renal function in the low and intermediate groups (Fig. 3b, c, Wilcoxon matched-pair signed-rank test). In the high group, renal function, which was decreased significantly 1 month after surgery, was improved significantly 1 year after surgery (Fig. 3d, *P* = 0.008, Wilcoxon matched-pairs signed rank test). As a result, renal function in the high group became comparable with the other groups 1 year after surgery (Fig. 4b *, P* = 0.8774, Mann–Whitney *U* test), which was significantly lower than that in the control group 1 month after surgery (Fig. 4a *, P* = 0.0448, Mann–Whitney *U* test). Using multivariate analysis, age (>65.5 years, OR 1.2, *P* = 0.03), male (OR 0.7, *P* = 0.03), postoperative eGFR <60 mL/min/1.72 m^2^ at 1 month (OR 9.0, *P* = 0.01) and operative duration >294 min (OR 6.2, *P* = 0.02) were selected as risk factors for significantly associated with eGFR <60 mL/min/1.72 m^2^ after radical cystectomy and orthotopic ileal neobladder construction, whereas presence of transient bilateral hydronephrosis (intermediate and high groups) was not selected (OR 0.4, *P* = 0.36, 95% CI 0.7–1.1) (Table 2).

**Table 2:** Univariate and multivariate logistic regression analyses of the risk factors for eGFR <60 mL/min/1.73 m^2^ at 1 year after surgery

Univariate	Risk factors	*P value*	Odds ratio	95% CI
Age	> 65.5 years	*0.11*	2.4	0.8–7.3
Gender	Male	*1.00*	1.0	0.3–3.4
Past history of				
Cardiovascular disease	Positive	*0.43*	0.4	0.0–3.8
Hypertension	Positive	*0.75*	0.8	0.3–2.6
Diabetes	Positive	*0.36*	1.9	0.5–8.2
Pathological T stage	pT3, 4	*0.68*	0.7	0.1–3.8
Pathological N status	pN+	*0.57*	2.3	0.1–38.1
Hydronephrosis at 1 M	> low	*0.22*	2.1	0.6–6.7
Neoadjuvant chemotherapy	received	*0.94*	0.9	0.1–10.6
Preoperative eGFR	< 60	*0.03*	7.0	1.2–40
Postoperative eGFR at 1 M	< 60	*0.00*	7.7	2.2–27
Complications (any grade)	Positive	*0.53*	1.5	0.4–4.8
Operative duration	> 294	*0.04*	3.4	1.1–10
Blood loss (g)	> 1380	*0.11*	2.4	0.8–7.3
Recurrence	Positive	*0.57*	2.3	0.1–38.1
Multivariate	Risk factors	*P value*	Odds ratio	95% CI
Age	> 65.5 years	*0.03*	1.2	1.1–1.5
Gender	Male	*0.03*	0.7	0.5–0.9
Hydronephrosis at 1 M	> low	*0.36*	0.4	0.7–1.1
Preoperative eGFR	< 60	*0.25*	3.7	0.4–34.0
Postoperative eGFR at 1 M	< 60	*0.01*	9.0	1.9–42.4
Operative duration	> 294	*0.02*	6.2	1.3–29.3

## Discussion

In the present study, we compared the grade of postoperative hydronephrosis to renal function at 1 month and 1 year after orthotopic ileal neobladder construction to assess the influence of postoperative transient hydronephrosis on renal function in patients with ileal neobladder construction. Our results suggest that transient postoperative hydronephrosis at 1 month had no significantly effect on renal function 1 year after ileal neobladder construction. Transient hydronephrosis may be caused by transient edema at anastomosis and reduced compliance of the neobladder. However, the significance and implication of postoperative hydronephrosis is controversial. Several previous studies reported that the postoperative decline in renal function and hydronephrosis were associated [4] and unassociated [2] after orthotopic ileal neobladder construction. Because most clinical reports describing renal function and postoperative hydronephrosis after orthotopic ileal neobladder construction are retrospective and cross-sectional, detailed information describing postoperative hydronephrosis are limited, and conclusions are impeded by differences in patient backgrounds, selection bias, and the surgical techniques used. Therefore, it is difficult to prove our hypothesis in the present study. Further prospective study is necessary to verify our findings.

In the present study, we developed original hydronephrosis grading system influencing on total renal function. We stratified patients into five categories (Grade 0 to 4) depending on hydronephrosis status on both sides (Fig. 1). Because the impact of unilateral grade 3 or 4 hydronephrosis on renal function remain unclear, we tried to make optimal stratification for unilateral grade 3 or 4 hydronephrosis. As a result, we found that unilateral grade 3 or 4 was suitable for the low group because these patients did not reduce renal function after urinary diversion. This might be due to the compensate recovery of contralateral kidney. However, it is unknown whether this hydronephrosis grading system is effective for other studies. Further studies with larger sample sizes are needed on this issue.

Radical cystectomy and urinary diversion remain the standard treatment modality for muscle-invasive bladder cancer patients. However, these are associated with the significant risks of perioperative and long-term morbidity and mortality [13, 14], including a subsequent decline in renal function [15–17]. The goals of urinary diversion after radical cystectomy have evolved from protecting the proximal portions of the tract to functional anatomical restoration because patients with urinary diversion are at a notably higher risk of decline in renal function [18–20]. In general, renal function is favorably preserved after continent urinary diversion compared with after conduit urinary diversion [21]; the incidence of a decline in renal function after continent urinary diversion has been reported range from 3 to 25% over 10 years [2, 4] In several reports, decline in renal function was observed immediately after radical cystectomy and urinary diversion, but stabilized afterward within 1–2 months [4, 17]. However, limited evidence is available describing the effects of orthotopic ileal neobladder construction on renal function after radical cystectomy. Several risk factors have been reported for the postoperative decline in renal function, including urinary tract obstruction, pyelonephritis, diabetes, and hypertension [1–4]. It is reasonable to suggest that urinary tract obstruction has a significant impact on the postoperative decline in renal function; however, the evidence is limited regarding the degree and critical duration of postoperative hydronephrosis for causing unrecoverable damage to renal function after orthotopic ileal neobladder construction.

An additional important factor for protecting the upper urinary tract is ureterointestinal anastomosis. A large number of techniques for ureterointestinal anastomosis have been described [21–23]. The most commonly used techniques for implantation into an ileal segment involve antirefluxing anastomoses (e.g., the afferent loop in the Studer pouch) [24], the Le Duc technique or use of a chimney [20], the split-cuff ureteric nipple [22]. and the serous-lined extramural tunnel [23]. However, comparisons between studies are challenging because of differences in patient age, underlying disorders, the use of radiotherapy, and preoperative/postoperative routines. To date, no single method has proved superior to others. Therefore, further studies are needed to address this issue.

This study has several limitations, including the small sample size, short follow-up periods, anastomosis techniques, its retrospective nature, and a sample composition that excluded many patients in whom CT imaging was not performed within 1 month. In addition, we were unable to control all variables, including selection bias, operative duration, influence of neoadjuvant chemotherapy, split renal function, continence status, uro-dynamic testing data, and other unmeasurable confounding factors. Statistical power was also insufficient due to the small sample size. Furthermore, long-term follow-up is necessary to address the long-term influences of transient hydronephrosis after orthotopic ileal neobladder construction, particularly in patients with severe bilateral hydronephrosis. Despite these limitations, this study was the first report to assess the influence of postoperative hydronephrosis on renal function at 1 month and 1 year after surgery. The data revealed no differences in postoperative renal function, regardless of the degree of postoperative transient hydronephrosis after radical cystectomy and orthotopic ileal neobladder construction.

## Conclusion

In conclusion, the presence of transient hydronephrosis immediately after surgery may have limited influence on renal function at 1 year after orthotopic ileal neobladder construction. Further investigation by well-designed randomized prospective studies is necessary to assess the influence between postoperative hydronephrosis and renal function in patients with orthotopic ileal neobladder construction.

## Abbreviations

CI: Confidence intervals
CT: Computed tomography
ECOG-PS: Eastern cooperative oncology group performance status
eGFR: Estimated glomerular filtration rate
ORs: Odds ratios
Q1: First quartile
Q3: Third quartile
